# Physiological evaluation of the Calgary adapted aRm ergometer (CARE) concussion exertion test in adolescent athletes: A repeated‐measures observational study

**DOI:** 10.1113/EP093264

**Published:** 2026-07-09

**Authors:** Joshua J. Burkart, Matthew G. Neill, Jean‐Michel Galarneau, Tegan Wilder, Sarah Johns, Joseph Carere, John J. Leddy, Mohammad N. Haider, William M. Adams, Cheri Blauwet, Chantel T. Debert, Carolyn A. Emery, Jonathan D. Smirl

**Affiliations:** ^1^ Cerebrovascular Concussion Laboratory, Faculty of Kinesiology University of Calgary Calgary Alberta Canada; ^2^ Sport Injury Prevention Research Centre, Faculty of Kinesiology University of Calgary Calgary Alberta Canada; ^3^ Human Performance Laboratory, Faculty of Kinesiology University of Calgary Calgary Alberta Canada; ^4^ Hotchkiss Brain Institute University of Calgary Calgary Alberta Canada; ^5^ Integrated Concussion Research Program University of Calgary Calgary Alberta Canada; ^6^ Alberta Children's Hospital Research Institute University of Calgary Calgary Alberta Canada; ^7^ Libin Cardiovascular Institute of Alberta University of Calgary Alberta Canada; ^8^ Cumming School of Medicine University of Calgary Calgary Alberta Canada; ^9^ UBMD Department of Orthopaedics and Sports Medicine, Jacobs School of Medicine and Biomedical Sciences SUNY at Buffalo Buffalo New York USA; ^10^ Adams Sports Medicine Consulting LLC Colorado Springs Colorado USA; ^11^ Department of Kinesiology University of North Carolina at Greensboro Greensboro North Carolina USA; ^12^ School of Sport, Exercise and Health Sciences, National Centre for Sport and Exercise Medicine Loughborough University Loughborough UK; ^13^ Department of Kinesiology Michigan State University East Lansing Michigan USA; ^14^ Department of Physical Medicine and Rehabilitation Spaulding Rehabilitation/Harvard Medical School Boston Massachusetts USA

**Keywords:** adolescence, arm crank ergometry, exercise is medicine, exertion testing, sport‐related concussion, transcranial doppler ultrasound

## Abstract

Exertion testing helps inform exercise prescription during concussion recovery. The Calgary Adapted aRm Ergometer (CARE) test, developed as the first upper‐body specific exertion test, aims to improve accessibility and inclusivity in concussion care. While initial development was conducted in adults, its applicability in adolescents remains unknown. This study compared physiological responses between the CARE test and the Calgary Concussion Cycle Test (CCCT) in non‐disabled adolescent male and female athletes. Fifteen females and fifteen males (aged 14–17) performed CARE and CCCT to volitional fatigue. Response differences, Bland–Altman plots, effect sizes, intraclass correlation and moderation by sex for heart rate (HR), middle cerebral artery velocity (MCAv), volume of oxygen consumption (${\dot{V}}_{O_{2}}$), minute ventilation (${\dot{V}}_{E}$) and end‐tidal carbon dioxide ($P_{\mathrm{ETC}O_{2}}$) were analysed at 25%, 50%, 75% and 100% of peak exertion levels. CARE elicited lower absolute values for HR, MCAv, $P_{\mathrm{ETC}O_{2}}$, ${\dot{V}}_{O_{2}}$ and ${\dot{V}}_{E}$ compared to CCCT, with differences and 95% limits‐of‐agreement becoming more pronounced at higher intensities. For mild‐to‐moderate exercise (25–50% exertion), the range typically used in clinical concussion testing, HR and MCAv differences were small for both sexes. Near maximal intensity, the HR gap widened, but MCAv was comparable. Sex did not modify the relationship between tests for HR and MCAv. While CARE predominantly employs smaller muscle mass than CCCT, results showed robust and relatively comparable physiological responses between tests and between male and female adolescents. These findings further support CARE as an accessible and inclusive post‐concussion exertion test for those not able to perform lower‐body protocols.

## Abstract (highlights)

- **What is the central question of this study?**
  How do the Calgary Adapted aRm Ergometer (CARE) concussion exertion test physiological and cerebrovascular responses compare to the Calgary Concussion Cycle Test (CCCT) in non‐disabled adolescent male and female athletes?
- **What is the main finding and its importance?**
  Despite large differences in muscle mass between the upper and lower body, the CARE test elicited smaller, but similar physiological responses to the CCCT for male and female adolescents at low‐to‐moderate intensities. These workloads are most relevant to post‐concussion exertion testing and support the CARE test as a viable, inclusive alternative for adolescents unable to perform traditional lower‐body post‐concussion exertion protocols (i.e., Para athletes, concurrent injury, modality preference).

## 1 INTRODUCTION

While sport and physical activity during adolescence offer critical benefits to physical, emotional, social and cognitive development (Brière et al., 2020), participation in these activities place adolescent athletes at risk of injury (Black et al., 2021). One of the most common injuries sustained by adolescent athletes is sport‐related concussion (SRC), with recent estimates suggesting 1 in 9 adolescents sustain an SRC each year (Black et al., 2021). Furthermore, adolescence represents a period of heightened susceptibility to concussion as the brain is undergoing significant neurodevelopment at this age (Murdaugh et al., 2018). While typical SRC recovery occurs around 4 weeks in adolescents, a minority subset (up to 30%) may go on to experience persisting symptoms after concussion (PSaC) (Broshek et al., 2022; Iuliano et al., 2026; Patricios et al., 2023; Zemek et al., 2017). Additionally, concussions present with heterogeneous symptoms and physiological disturbances (Patricios et al., 2023). Given the diversity of SRC presentation and the potential for prolonged recovery, best‐practice recommendations emphasise a multidisciplinary team approach to concussion care including assessments of autonomic, vestibular, ocular and cervical systems and exercise tolerance (Patricios et al., 2023).

As part of the multifaceted approach to concussion care, graded aerobic exertion testing has emerged as a valuable clinical tool to assess concussion symptomology and provide exercise prescriptions post‐injury (Patricios et al., 2023). Current return‐to‐sport/school/work recommendations suggest a relative rest period of 1–2 days, followed by a gradual return to these activities, which is aided when informed exercise prescriptions are employed (Haider et al., 2021; Leddy et al., 2018, 2023). Clinically, the purpose of exertion testing post‐concussion is to elicit increases in physiological responses (i.e., heart rate (HR), blood pressure, brain blood flow, ventilation) and examine how individuals respond across various clinical symptom profiles to help improve recovery trajectories (Haider et al., 2021; Leddy et al., 2018). While a mild exacerbation of clinical symptoms is expected during the recovery period from concussion, if an increase of ≥3 points on a 0–10 visual analogue scale is observed, the individual is deemed to be exercise intolerant (Leddy et al., 2018). This threshold is subsequently used to inform individualised sub‐symptom‐threshold exercise prescriptions to promote recovery (Haider et al., 2021; Leddy et al., 2018). In adolescents, it has been shown females who are exercise intolerant have a greater likelihood of experiencing a delayed recovery (Neill et al., 2025). Furthermore, early engagement in sub‐threshold aerobic exercise following concussion has been shown to shorten recovery duration and reduce the likelihood of PSaC (Leddy et al., 2023; Mn et al., 2021).

Several validated protocols exist to assess exercise (in)tolerance following SRC in adolescents, including the Buffalo Concussion Treadmill Test (BCTT) (Leddy & Willer, 2013) and the Buffalo Concussion Bike Test (BCBT) (Haider et al., 2019). More recently, the Calgary Concussion Cycling Test (CCCT) was validated in adults against the BCTT, comparing a more in‐depth physiological profile between the two tests due to the known impact of concussion on functional responses beyond HR (Miutz et al., 2023). While these protocols have clinical value, they rely on lower‐body exercise, limiting accessibility for individuals with concurrent lower‐extremity injuries, mobility impairments, or for sports emphasising upper‐body function (e.g., Para sport). In response to this gap, the Calgary Adapted aRm Ergometer (CARE) test was developed as the first upper‐body specific post‐concussion exertion protocol and was compared to the CCCT in healthy, non‐disabled adults (Smirl et al., 2026). The CARE test offers a standardised and graded upper‐body protocol using arm crank ergometry (ACE) to elicit increases in cardiovascular, cerebrovascular and respiratory responses (Smirl et al., 2026). In adults, ACE yields lower peak HR, oxygen consumption and ventilation, but imposes higher relative physiological strain at equivalent workloads due to reduced active muscle mass (Larsen et al., 2016). Whether similar responses occur in adolescents, particularly in the context of post‐concussion testing, remains unknown.

Additionally, sex and gender‐based differences in concussion outcomes are increasingly recognised in adolescent populations (Frommer et al., 2011; Koerte et al., 2020). Girls often report greater symptom burden and more functional impairments than boys (Koerte et al., 2020). Female adolescents are also at potentially greater risk of exercise intolerance (Neill et al., 2025), which may be related to differences in autonomic and cerebrovascular responses during post‐concussion exertion testing (Ashley et al., 2020; Neill et al., 2024). For example, while cerebral blood flow (CBF) declines from childhood into adolescence, a sex divergence emerges during puberty where CBF increases in females and continues to decline in males (Satterthwaite et al., 2014). Post‐pubertal females also demonstrate greater exercise‐related increases in CBF (Douglas et al., 2024), whereas adolescent males tend to exhibit stronger cerebrovascular autoregulation (Vavilala et al., 2005). Despite these known sex‐based physiological differences between adolescent males and females, few studies have explored how they may influence responses to post‐concussion exertion protocols. Accordingly, the objective of this study was to compare cardiovascular, cerebrovascular and respiratory responses between the CARE test and the CCCT in healthy, non‐disabled male and female adolescent athletes. It was hypothesised that the CARE test would elicit robust physiological responses comparable to the CCCT in both sexes, with physiological differences becoming more pronounced as relative workload increased. Further, we hypothesised agreement between CARE and CCCT would be comparable between male and female participants.

## 2 METHODS

### 2.1 Ethical approval

All methodology in the current investigation was approved by the University of Calgary's Conjoint Health and Research Ethics Board (REB21‐1517). All study procedures and protocols adhered to the guidelines highlighted in the *Declaration of Helsinki* (2013 revision, except for study registration in a database) (World Medical Association, 2013). All participant questions were addressed, written informed consent and parental consent (if necessary) were gathered, and a Physical Activity Readiness Questionnaire (PARQ+) was completed prior to data collection.

### 2.2 Participants and study design

Based on previous studies (Haider et al., 2019; Leddy & Willer, 2013; Miutz et al., 2023; Smirl et al., 2026), the current investigation recruited 30 (15 female and 15 male) healthy uninjured and non‐disabled adolescent athletes between the ages of 14 and 17 to visit the laboratory for two exercise testing visits. Participants in this study were asked to self‐report both biological sex and gender identity, with all participants identifying as *cis*‐gender. Accordingly, data in the current study were analysed and discussed in reference to biological sex. Participants were excluded if they had sustained a concussion within the past 6 months and/or had the presence of any cardiovascular, musculoskeletal, neurological or cerebrovascular condition(s) impairing participants’ ability to perform maximal intensity exercise (Churchill et al., 2019; Kamins et al., 2017; Lapointe et al., 2021). Based upon the above criteria, none of the recruited participants were excluded from study participation. Participants were instructed to abstain from caffeine, alcohol, vaping, smoking and heavy exercise for a minimum of 8 h prior to each of the two testing sessions as these variables are known to confound the response of the cardiovascular and cerebrovascular systems to exercise (Ainslie et al., 2007; Burma et al., 2021; Burma, Copeland et al., 2020, 2020). Many studies investigating vascular outcomes in males and females restrict participation to the early follicular phase to control for the acute, vasoprotective effects of oestrogen and progesterone on vascular function (Stachenfeld & Taylor, 2014). However, it has been argued that this method ‘puts the cart before the horse’, as it limits sex comparisons of vascular function to a particular phase of the menstrual cycle, likely biasing findings towards an absence of sex differences and failing to generalise to females broadly (Giersch et al., 2020). Thus, while there is a present need to understand how fluctuating sex hormones influence sex differences in concussion pathophysiology, the present study opted to include female participants regardless of menstrual status or phase, to maximise clinical generalisability.

### 2.3 Experimental protocols

Data collection was conducted at the Cerebrovascular Concussion Laboratory, University of Calgary, situated 1111 m above sea level. Adolescent participants visited the laboratory on two occasions where they completed both the CARE and CCCT tests to volitional fatigue (order of tests randomly assigned). The two exercise testing visits were separated by a minimum of 1 day (median: 6 days, interquartile range: 3–14 days). Sex, gender identity, age, height and weight for each participant were recorded. Body mass index (BMI) was calculated as the weight in kilograms divided by the squared height in metres for each participant.

### 2.4 Calgary Concussion Cycle Test

The CCCT (Miutz et al., 2023) was completed on a stationary cycle ergometer (Lode Cycle Ergometer, Corival cpet model, Groningen, Netherlands). To account for the relative muscle mass differences between males and females, the CCCT employs sex‐specific formulas to determine individual wattage increases. As per the CCCT protocol, the starting wattage is determined to be 0.14 × kilogram of body mass for males, and 0.11 × kilogram of body mass in females (Miutz et al., 2023). Each subsequent stage of the CCCT (1 min in duration) increases by the same individualised wattage until participants attain volitional fatigue and cannot maintain a cadence between 70 and 90 rpm. Participants self‐adjusted the seat height to a position that allowed for comfortable full‐leg extension during cycling.

### 2.5 CARE test

The CARE test was performed on an arm crank ergometer (Lode ACE, Angio CPET model) (Smirl et al., 2026). The starting wattage was calculated as 1 W per 3 kg (6.7 pounds) of the participant's 10‐repetition maximum bicep curl, with the workload increasing by the same increment every minute until volitional fatigue (Smirl et al., 2026). Participants were seated such that the centre of the crankshaft aligned horizontally with the shoulder joint. The seat position was self‐adjusted to ensure a slight bend in the elbow when the arm was fully extended. Participants were asked to sit fully back in the chair throughout the test, keeping their feet flat on the floor and positioned in front of them to maintain consistent posture. Given the known positive influence of arm cranking cadence on peak HR, ventilation and oxygen consumption, participants were instructed to maintain a cadence between 70 and 90 rpm during testing (Smith et al., 2001). A warm‐up stage was not conducted prior to either exercise test, as both the CARE test and CCCT employ a low initial workload and gradual stage progression designed for post‐concussion application. To compare between the CCCT and CARE tests as they were performed at different absolute wattage increases, the normalised percentage for the completion of each test was used as all individuals reported reaching a maximal RPE Borg rating score of 20 on both tests. For further details of the specifics between CCCT and CARE test comparisons, refer to the statistical analysis section of the manuscript.

### 2.6 Instrumentation

During each data collection setup, participants’ middle cerebral arteries (MCA) were insonated bilaterally using transcranial doppler (TCD) ultrasound (DWL USA, Inc., San Juan Capistrano, CA, USA). MCA velocity (MCAv) was used as an index of CBF due to its temporal superiority and reliability during exercise testing (Skow et al., 2022). Two 2‐MHz TCD probes were positioned at the transtemporal window and fitted into place with an adjustable headframe (DWL USA). Vessel insonation was performed by highly trained sonographers who confirmed proper insonation of left and right MCA with carotid compressions (Willie et al., 2011). Left and right MCAv were verified to be within 10% prior to testing with vessel depths and velocities recorded for each participant during their first testing session to be used as reference values for the second testing session to maximise between‐day reliability of MCAv assessment (Smirl et al., 2015). Continuous HR measurement was captured with a POLAR HR monitor (POLAR H10, Kempele, Finland). Finger photoplethysmography (Finometer NOVA; Finapres Medical Systems, Amsterdam, The Netherlands) was used to record beat‐to‐beat mean arterial pressure (MAP) corrected for differences in hand height relative to the heart with a height correction unit. To limit the signal interference during exercise, participants were instructed to avoid squeezing the handles of the ergometers with the finger the cuff was situated on. Minute ventilation (${\dot{V}}_{E}$), volume of oxygen consumption (${\dot{V}}_{O_{2}}$) (mixing chamber) and partial pressure of end‐tidal carbon dioxide ($P_{\mathrm{ETC}O_{2}}$) (breath‐by‐breath) were measured using a mouthpiece, pneumotachograph, nose clip and two separate inline gas analysers (ML206; ADInstruments, Colorado Springs, CO, USA). Both gas analysers and the pneumotachograph were calibrated prior to testing with two‐point calibration (room air: 78% nitrogen, 21% oxygen and 0.04% carbon dioxide; known gas concentration: 5% carbon dioxide and 16% oxygen) and a 3‐L syringe respectively. MCAv, HR, MAP, ${\dot{V}}_{E}$, ${\dot{V}}_{O_{2}}$ and $P_{\mathrm{ETC}O_{2}}$ data were collected at 1000 Hz and stored offline for analysis using commercially available software (LabChart Pro Version 8, ADInstruments). Finally, at the end of each CARE and CCCT stage, participants rating of perceived exertion (RPE) was recorded with the Borg 6–20 scale (Borg, 1982).

### 2.7 Estimation of pubertal status

The heights of each participant's biological parents were recorded via self‐report for use in the estimation of each participants’ pubertal status assessed using the Khamis–Roche method (percentage of adult height attained (PAH)) (Khamis & Roche, 1994) and the modified Moore maturity offset equation (years since peak‐height velocity (PHV)) (Moore et al., 2015). Using the Khamis–Roche method, 6 of the 30 adolescent participants were classified as pubertal (88–95% PAH) and 24 as post‐pubertal (>95% PAH) (Khamis & Roche, 1994). All participants’ classified as pubertal were male. Using the modified Moore equation with a ±0.5‐year threshold to define ‘circa‐PHV’ status, two participants (both male) were classified as circa‐PHV and 28 as post‐PHV (Moore et al., 2015). Notably, both methods classified all female participants as post‐pubertal or post‐PHV, aligning with sex‐specific patterns of pubertal timing, where females tend to experience peak height velocity earlier than males (Rogol et al., 2000). Due to the small number of adolescent participants classified as pubertal/circa‐PHV, the present study is not adequately powered to consider the potential influence of estimated pubertal status on study results.

### 2.8 Data processing

For each physiological variable, a mean of datapoints occurring within the final 20 s of each stage (60 s) of both the CARE test and CCCT were extracted in LabChart Pro for use in the statistical analyses. Although MAP was initially intended to be assessed using finger photoplethysmography, usable data could not be obtained due to persistent signal degradation, excessive motion artifact and poor participant tolerance of the finger cuff (Castaneda et al., 2018). These challenges were particularly evident during the CARE test, where continuous hand and finger movement greatly impaired signal quality. Given these limitations and the known limitation of assessing blood pressure during arm cranking (Hollingsworth et al., 1990), MAP data were excluded from the final analysis.

### 2.9 Statistical analysis

**FIGURE 1:**
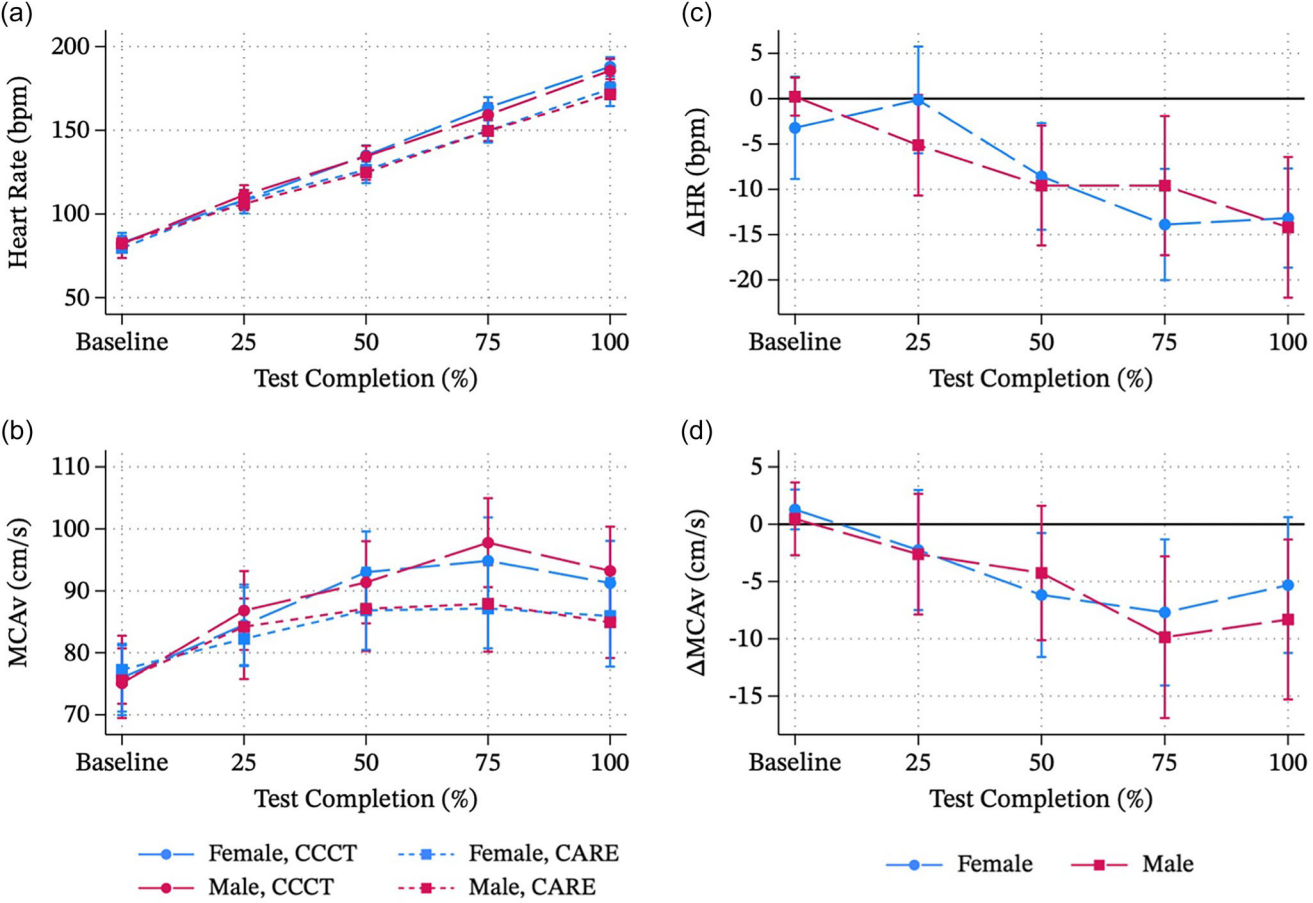
(a, b) Comparison of HR (a) and MCAv (b) responses by sex during the CCCT and the CARE test performed to volitional fatigue for 15 male and 15 female adolescent participants (aged 14–17) who completed both exertional testing sessions. Data are presented as means ± 95% CI. (c, d) Mean differences by sex between the CCCT and CARE test for HR (c) and MCAv (d). CARE test, Calgary Adapted aRm Ergometer Test; CCCT, Calgary Concussion Cycle Test; HR, heart rate; MCAv, middle cerebral artery blood velocity.

**FIGURE 2:**
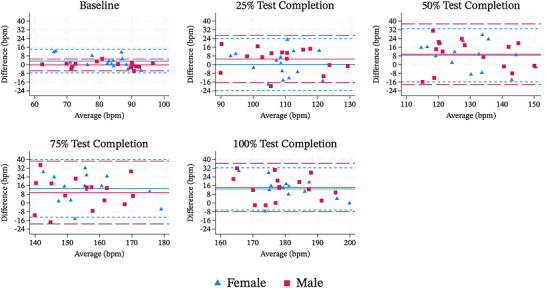
Bland–Altman plots with mean bias and 95% limits of agreement by sex for heart rate (HR) (bpm) between the Calgary Concussion Cycle Test and the Calgary Adapted aRm Ergometer (CARE) test from 15 female and 15 male adolescent athletes (aged 14–17). Adolescent females are represented by the blue triangles, while adolescent males are represented by the red squares. Adolescent females mean bias and 95% limits of agreement represented by blue line and blue dashed lines, respectively. Adolescent males mean bias and 95% limits of agreement represented by red line and red dashed lines, respectively.

**FIGURE 3:**
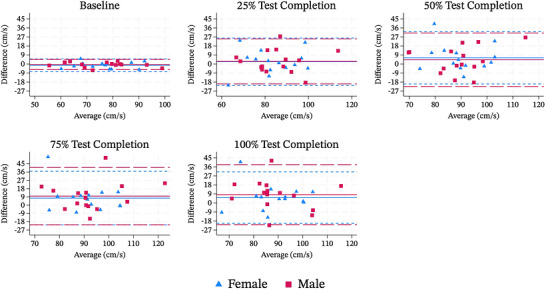
Bland–Altman plots with mean bias and 95% limits of agreement by sex for middle cerebral artery velocity (MCAv) (cm/s) between the Calgary Concussion Cycle Test and the Calgary Adapted aRm Ergometer (CARE) test from 15 female and 15 male adolescent athletes (aged 14–17). Adolescent females are represented by the blue triangles, while adolescent males are represented by the red squares. Adolescent females mean bias and 95% limits of agreement represented by blue line and blue dashed lines, respectively. Adolescent males mean bias and 95% limits of agreement represented by red line and red dashed lines, respectively.

**FIGURE 4:**
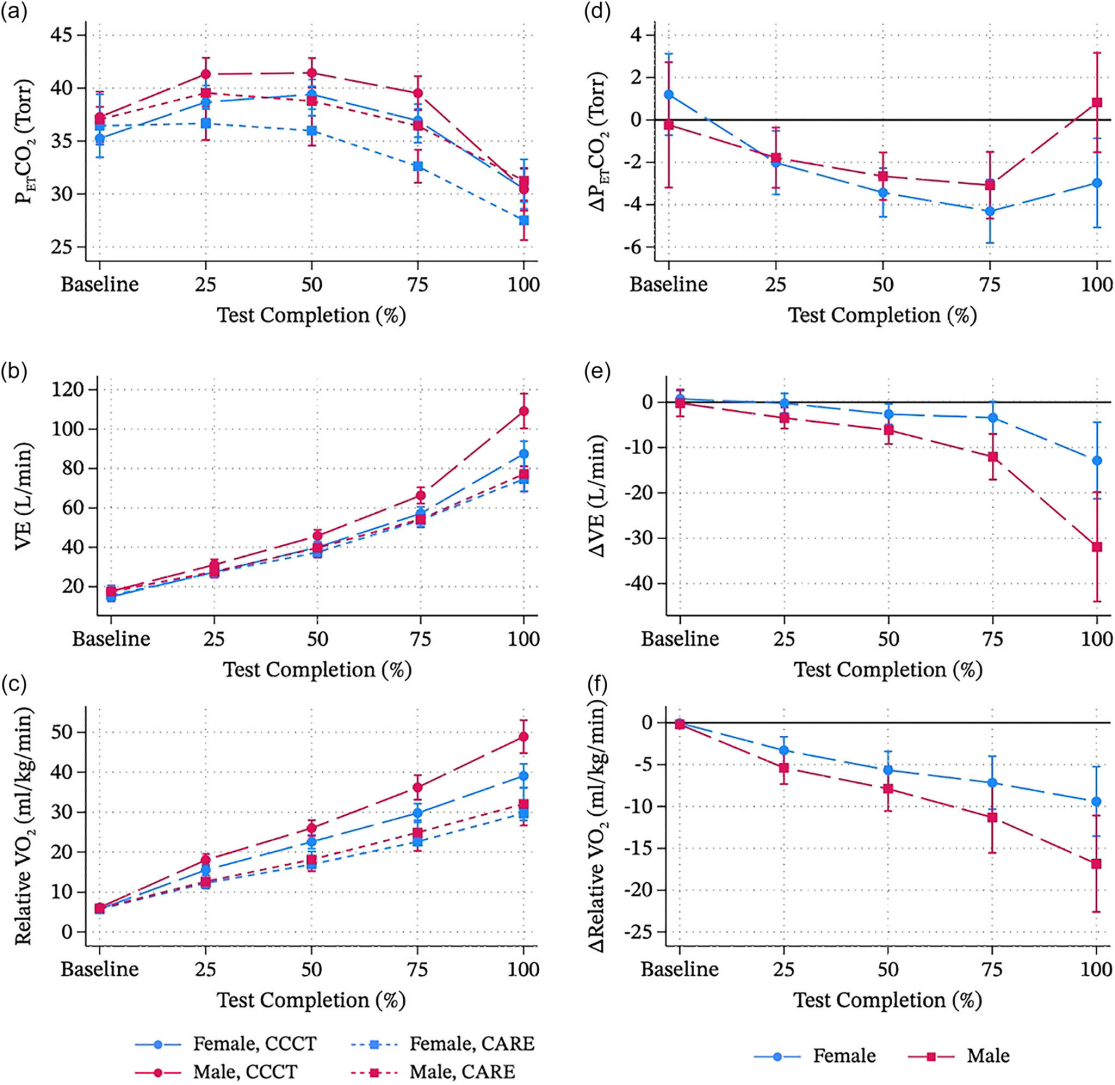
(a–c) Comparison of $P_{\mathrm{ETC}O_{2}}$(a), ${\dot{V}}_{E}$ (b) and ${\dot{V}}_{O_{2}}$ (c) responses by sex during the CCCT and the CARE test performed to volitional fatigue for 15 male and 15 female adolescent participants (aged 14–17) who completed both exertional testing sessions. Data are presented as means ± 95% CI. (d–f) Mean differences by sex between the CCCT and CARE test for $P_{\mathrm{ETC}O_{2}}$(d), ${\dot{V}}_{E}$ (e) and ${\dot{V}}_{O_{2}}$(f). CARE test, Calgary Adapted aRm Ergometer Test; CCCT, Calgary Concussion Cycle Test; $P_{\mathrm{ETC}O_{2}}$, partial pressure of end‐tidal carbon dioxide; ${\dot{V}}_{E}$, minute ventilation; ${\dot{V}}_{O_{2}}$, volume of oxygen consumption.

**FIGURE 5:**
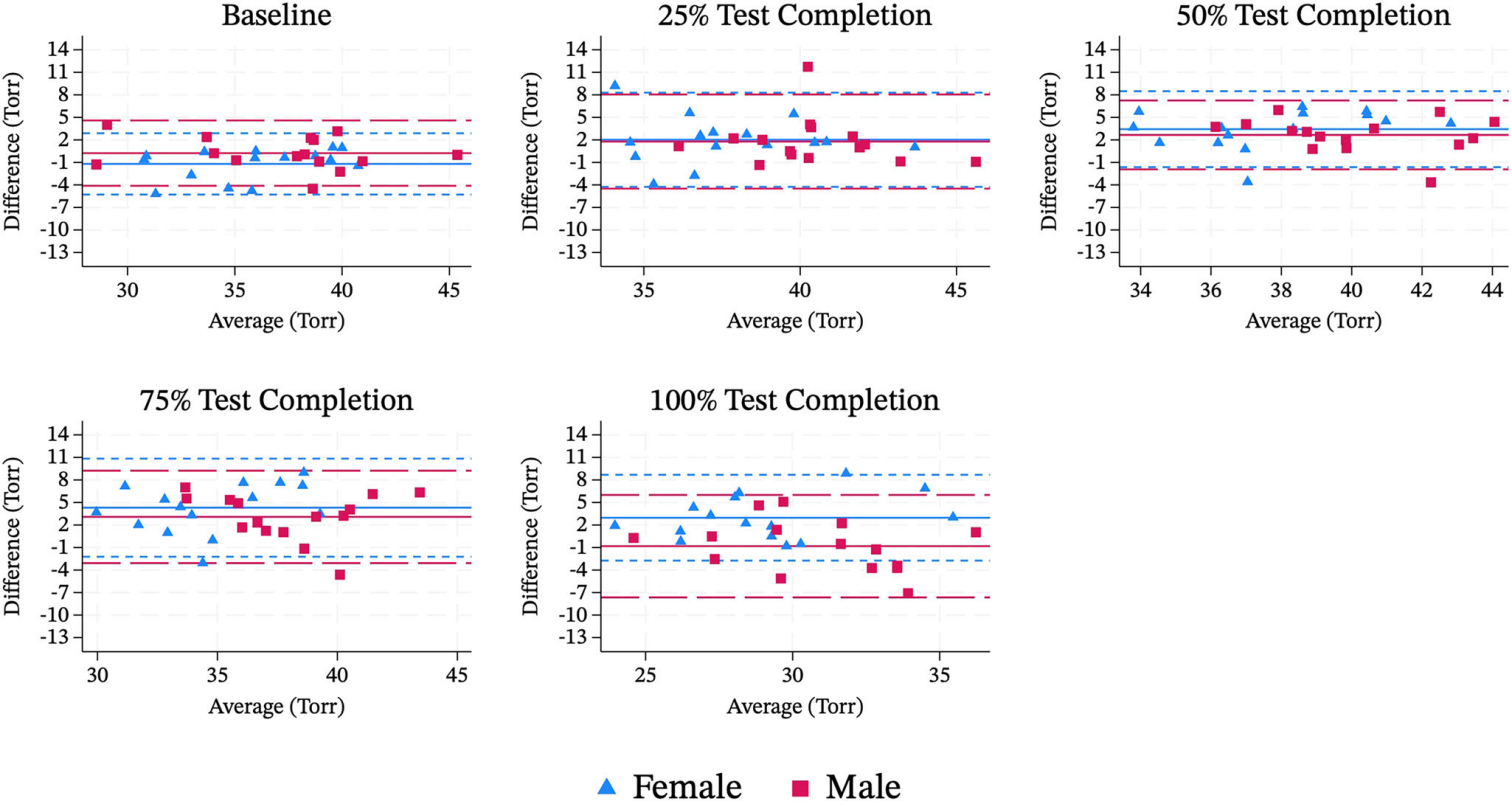
Bland–Altman plots with mean bias and 95% limits of agreement by sex for partial pressure of end‐tidal carbon dioxide ($P_{\mathrm{ETC}O_{2}}$, Torr) between the Calgary Concussion Cycle Test and the Calgary Adapted aRm Ergometer (CARE) test from 15 female and 15 male adolescent athletes (aged 14–17). Adolescent females are represented by the blue triangles, while adolescent males are represented by the red squares. Adolescent females mean bias and 95% limits of agreement represented by blue line and blue dashed lines, respectively. Adolescent males mean bias and 95% limits of agreement represented by red line and red dashed lines, respectively.

**FIGURE 6:**
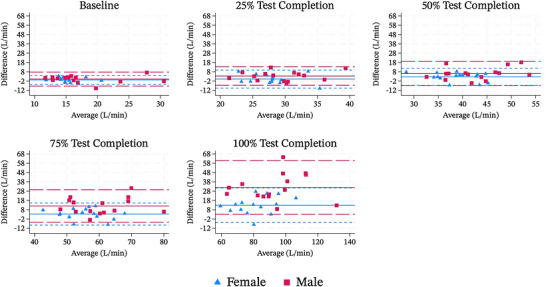
Bland–Altman plots with mean bias and 95% limits of agreement by sex for minute ventilation (${\dot{V}}_{E}$) (L/min) between the Calgary Concussion Cycle Test and the Calgary Adapted aRm Ergometer (CARE) test from 15 female and 15 male adolescent athletes (aged 14–17). Adolescent females are represented by the blue triangles, while adolescent males are represented by the red squares. Adolescent females mean bias and 95% limits of agreement represented by blue line and blue dashed lines, respectively. Adolescent males mean bias and 95% limits of agreement represented by red line and red dashed lines, respectively.

**FIGURE 7:**
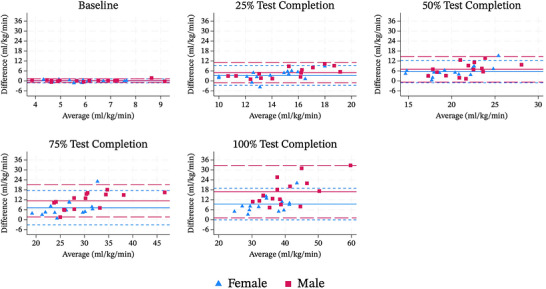
Bland–Altman plots with mean bias and 95% limits of agreement by sex for relative volume of oxygen consumption (${\dot{V}}_{O_{2}}$) (ml/kg/min) between the Calgary Concussion Cycle Test and the Calgary Adapted aRm Ergometer (CARE) test from 15 female and 15 male adolescent athletes (aged 14–17). Adolescent females are represented by the blue triangles, while adolescent males are represented by the red squares. Adolescent females mean bias and 95% limits of agreement represented by blue line and blue dashed lines, respectively. Adolescent males mean bias and 95% limits of agreement represented by red line and red dashed lines, respectively.

All statistical analyses were performed in Stata (release 19, 2025; StataCorp LLC, College Station, TX, USA) by a biostatistician (J.M.G.). To account for intensity and individual differences in test duration between the CARE and CCCT protocols, all physiological parameters were normalised to percentage completion of each test as all individuals reported reaching a maximal RPE Borg rating score of 20 on both tests. Accordingly, mean values were compared across five relative intensity time points: baseline, 25%, 50%, 75% and 100% of test completion (Figures 1 and 4). A multilevel linear mixed‐effects model was employed for each physiological outcome (HR, MCAv, $P_{\mathrm{ETC}O_{2}}$, ${\dot{V}}_{E}$ and relative ${\dot{V}}_{O_{2}}$), including fixed effects for sex, condition (CARE vs. CCCT) and stage, in a three‐way interaction (full factorial). Heteroskedastic residuals were produced by sex and stage combination to produce sex by stage specific intraclass correlation coefficients (ICCs) (Galarneau et al., 2026), and models were fitted using restricted maximum likelihood with Kenward–Roger degrees of freedom to account for the modest sample size (Rabe‐Hesketh & Skrondal, 2022). Although a single model was used per outcome, sex‐specific results were extracted using linear combinations of beta coefficients. For each sex and stage, means and 95% confidence intervals (CI) were produced for the CARE and CCCT with corresponding mean differences (Tables 2 and 3) to examine mean differences between both modalities by stage but also to investigate the potential moderating effects that sex might have had on these mean differences. Furthermore, ICCs and Cohen's *d* effect sizes were derived at each stage within sex (Tables 4 and 5). Additionally, to further explore the potential for moderation by sex, differences in ICCs between sexes were formally tested using nonlinear‐combinations of parameters with standard errors computed using the delta method (https://www.stata.com/manuals/meestaticc.pdf) (Tables 4 and 5). Finally, examination of differences and individual variability between modalities was made through the use of Bland–Altman plots color‐coded with 95% limits of agreement (LoA) by sex (Figures 2, 3 and 5, 6, 7). Following best practices in physiological research, interpretation was based on a combination of 95% CI, 95% LoA, ICC, effect size and *P*‐values (set a priori at *P *< 0.05 for significance) rather than dichotomous significance thresholds (Amrhein et al., 2019). ICCs were interpreted as excellent (>0.90), good (0.75–0.90), moderate (0.50–0.75) or poor (<0.50) (Koo & Li, 2016) and Cohen's *d* effect sizes as negligible (<0.20), small (0.20–0.49), moderate (0.50–0.79), or large (≥0.80) (Lakens, 2013).

## 3 RESULTS

Thirty healthy, non‐disabled adolescent athletes (15 female,15 male) completed both exercise protocols (CARE and CCCT) and were included in the analysis. Demographic characteristics are presented in Table 1. The mean number of stages completed in the CCCT was 23 ± 4 (females, 25 ± 5; males, 22 ± 3) and 22 ± 4 (females, 22 ± 3; males, 22 ± 4) in CARE (Table 1). All participants reached an RPE of 20 during the final stage of testing (100% of test completion) on both CARE and CCCT. At baseline, no significant differences in physiological values were observed between protocols for either males or females (Tables 2 and 3). Additionally, sex did not significantly moderate the relationship between protocols at baseline for any of the outcomes (Tables 2 and 3).

**TABLE 1:** Sex stratified characteristics for adolescent participants (aged 14–17) who completed both the CARE test and CCCT (*n* = 30, 15F:15M)

	Participant characteristics				CARE		CCCT	
Sample	Age (years)	Height (cm)	Weight (kg)	BMI (kg/m^2^)	Wattage increase	Stages completed	Wattage increase	Stages completed
**Female (*n* = 15)**	16.1 (0.9)	166.9 (6.0)	63.1 (9.3)	22.6 (2.5)	3 (1)	22 (3)	7 (1)	25 (5)
**Male (*n* = 15)**	15.3 (1.2)	176 (7.1)	68.4 (12.8)	21.9 (3.1)	4 (1)	22 (4)	10 (2)	22 (3)

*Note*: Data presented as means (SD). The table also displays mean wattage increase and number of stages completed for both the CARE test and CCCT. Abbreviations: CARE test, Calgary Adapted aRm Ergometer Test; CCCT, Calgary Concussion Cycle Test.

**TABLE 2:** Mean values and mean differences by sex, and analysis of sex moderation for HR and MCAv for 30 adolescent participants (15F:15M, aged 14–17) who completed both the CARE test and CCCT

		Male										Female													
		CCCT			CARE			Mean difference				CCCT			CARE			Mean difference				Sex moderation			
Parameter	Comparison	Mean	95% lower	95% upper	Mean	95% lower	95% upper	Mean difference	95% lower	95% upper	*P*	Mean	95% lower	95% upper	Mean	95% lower	95% upper	Mean difference	95% lower	95% upper	*P*	DiD	95% lower	95% upper	*P*
**HR (bpm)**	**Baseline**	82	78	87	82	78	87	0	−2	2	0.833	83	77	89	80	74	86	3	−2	9	0.263	−3	−9	3	0.262
	**25%**	111	105	117	106	100	112	5	0	11	0.070	108	102	114	108	102	114	0	−6	6	0.962	5	−3	13	0.228
	**50%**	134	128	141	125	118	131	10	3	16	0.005^*^	135	129	141	126	120	132	9	3	14	0.004^*^	1	−8	10	0.823
	**75%**	159	152	166	150	143	157	10	2	17	0.014^*^	164	158	170	150	144	156	14	8	20	<0.001^*^	−4	−14	6	0.392
	**100%**	186	179	193	171	164	178	14	6	22	<0.001^*^	188	182	194	175	169	181	13	8	19	<0.001^*^	1	−8	11	0.832
**MCAv (cm/s)**	**Baseline**	75.1	69.5	80.7	75.6	69.9	81.2	−0.5	−3.7	2.7	0.770	76.0	70.5	81.5	77.3	71.8	82.8	−1.3	−3.0	0.5	0.146	0.8	−2.8	4.4	0.660
	**25%**	86.8	80.5	93.2	84.2	77.8	90.6	2.6	−2.7	7.9	0.331	84.5	78.0	91.0	82.3	75.8	88.8	2.3	−3.0	7.5	0.400	0.4	−7.1	7.8	0.924
	**50%**	91.4	84.8	98.0	87.1	80.5	93.7	4.3	−1.6	10.1	0.156	93.0	86.4	99.6	86.8	80.3	93.4	6.2	0.8	11.6	0.025^*^	−1.9	−9.9	6.1	0.637
	**75%**	97.8	90.6	105.0	87.9	80.7	95.1	9.9	2.8	16.9	0.006^*^	94.9	87.9	101.9	87.2	80.2	94.2	7.7	1.3	14.1	0.018^*^	2.2	−7.3	11.7	0.655
	**100%**	93.2	86.1	100.4	84.9	77.8	92.1	8.3	1.3	15.3	0.020^*^	91.3	84.5	98.1	85.9	79.2	92.7	5.3	−0.6	11.2	0.079	3.0	−6.2	12.2	0.521

Data were normalised to percentage of test completion to account for variations in test duration between the CARE test and CCCT. ^*^Significance (*P *< 0.05). Abbreviations: CARE test, Calgary Adapted aRm Ergometer Test; CCCT, Calgary Concussion Cycle Test; DiD, difference in differences; HR, heart rate, MCAv, middle cerebral artery blood velocity.

**TABLE 3:** Mean values and mean differences by sex, and analysis of sex moderation for $P_{\mathrm{ETC}O_{2}}$, ${\dot{V}}_{E}$ and relative ${\dot{V}}_{O_{2}}$ for 30 adolescent participants (15F:15M, aged 14–17) who completed both the CARE test and CCCT

		Male										Female													
		CCCT			CARE			Mean difference				CCCT			CARE			Mean difference				Sex moderation			
Parameter	Comparison	Mean	95% lower	95% upper	Mean	95% lower	95% upper	Mean difference	95% lower	95% upper	*P*	Mean	95% lower	95% upper	Mean	95% lower	95% upper	Mean difference	95% lower	95% upper	*P*	DiD	95% lower	95% upper	*P*
$P_{\mathrm{ETC}O_{2}}$ **(Torr)**	**Baseline**	37.3	34.9	39.7	37.0	34.7	39.4	0.2	−2.7	3.2	0.877	35.3	33.5	37.0	36.5	34.7	38.2	−1.2	−3.1	0.7	0.222	1.4	−2.1	5.0	0.426
	**25%**	41.3	39.8	42.9	39.5	38.0	41.1	1.8	0.4	3.2	0.014^*^	38.7	37.1	40.2	36.7	35.1	38.2	2.0	0.5	3.5	0.009^*^	−0.2	−2.3	1.8	0.823
	**50%**	41.4	40.0	42.8	38.8	37.4	40.2	2.7	1.5	3.8	<0.001^*^	39.4	38.0	40.8	36.0	34.6	37.4	3.4	2.3	4.6	<0.001^*^	−0.8	−2.4	0.8	0.347
	**75%**	39.5	37.9	41.1	36.4	34.8	38.0	3.1	1.5	4.7	<0.001^*^	36.9	35.4	38.5	32.6	31.1	34.2	4.3	2.8	5.8	<0.001^*^	−1.2	−3.4	0.9	0.267
	**100%**	30.5	28.4	32.5	31.3	29.3	33.3	−0.8	−3.2	1.5	0.493	30.5	28.6	32.4	27.5	25.7	29.4	3.0	0.9	5.1	0.006^*^	−3.8	−6.9	−0.6	0.018^*^
${\dot{V}}_{E}$ **(L/min)**	**Baseline**	17.7	14.7	20.7	17.5	14.5	20.5	0.2	−2.8	3.2	0.894	14.9	12.4	17.4	15.6	13.1	18.1	−0.7	−2.5	1.1	0.433	0.9	−2.5	4.4	0.604
	**25%**	31.1	28.4	33.8	27.6	25.0	30.3	3.5	1.2	5.8	0.003^*^	27.4	24.8	30.0	27.2	24.6	29.8	0.2	−1.9	2.4	0.839	3.3	0.1	6.4	0.043^*^
	**50%**	45.8	42.8	48.8	39.6	36.6	42.7	6.1	3.1	9.2	<0.001^*^	40.0	37.4	42.7	37.4	34.7	40.1	2.6	0.4	4.9	0.023^*^	3.5	−0.3	7.3	0.071
	**75%**	66.3	62.2	70.4	54.3	50.1	58.4	12.0	7.0	17.1	<0.001^*^	57.2	53.9	60.5	53.8	50.4	57.1	3.4	−0.2	7.0	0.064	8.6	2.4	14.8	0.006^*^
	**100%**	109.1	100.4	117.9	77.2	68.4	86.0	31.9	19.9	44.0	<0.001^*^	87.5	81.2	93.8	74.6	68.3	81.0	12.9	4.4	21.3	0.003^*^	19.1	4.3	33.8	0.011^*^
**Relative** ${\dot{V}}_{O_{2}}$ **(ml/kg/min)**	**Baseline**	6.2	5.5	6.9	6.0	5.2	6.7	0.2	−0.1	0.5	0.169	5.7	5.0	6.5	5.7	5.0	6.4	0.0	−0.3	0.4	0.853	0.2	−0.3	0.6	0.448
	**25%**	18.0	16.5	19.5	12.7	11.1	14.2	5.4	3.4	7.3	<0.001^*^	15.5	14.2	16.9	12.2	10.9	13.6	3.3	1.7	4.9	<0.001^*^	2.1	−0.5	4.6	0.107
	**50%**	26.0	24.1	28.0	18.2	16.1	20.2	7.9	5.2	10.6	<0.001^*^	22.6	20.9	24.3	16.9	15.2	18.7	5.7	3.4	7.9	<0.001^*^	2.2	−1.3	5.7	0.210
	**75%**	36.2	33.1	39.3	24.9	21.8	28.0	11.3	7.1	15.5	<0.001^*^	29.8	27.5	32.1	22.6	20.3	25.0	7.2	4.0	10.3	<0.001^*^	4.1	−1.2	9.4	0.125
	**100%**	48.9	44.8	53.0	32.1	28.0	36.2	16.8	11.1	22.6	<0.001^*^	39.1	36.1	42.1	29.7	26.7	32.7	9.4	5.2	13.5	<0.001^*^	7.4	0.4	14.5	0.040^*^

Data were normalised to percentage of test completion to account for variations in test duration between the CARE test and CCCT. ^*^Significance (*P *< 0.05). Abbreviations: CARE test, Calgary Adapted aRm Ergometer Test; CCCT, Calgary Concussion Cycle Test; DiD, difference in differences; $P_{\mathrm{ETC}O_{2}}$, partial pressure of end‐tidal carbon dioxide; ${\dot{V}}_{O_{2}}$, volume of oxygen consumption; ${\dot{V}}_{E}$, minute ventilation.

### 3.1 Cardiovascular and cerebrovascular responses (HR and MCAv)

HR increased progressively during the test for both males and females for the CCCT and CARE protocols (Figure 1). For males, HR was consistently higher on the CCCT than CARE, with mean differences ranging from 5 bpm (95% CI: 0, 11) at 25% to 14 bpm (95% CI: 6, 22) at 100% test completion (Table 2). Effect sizes increased from small to large as test completion progressed, while ICCs ranged from moderate to poor (Table 4). Among females, HR differences increased from 0 bpm (95% CI: –6, 6) at 25% to 13 bpm (95% CI: 8, 19) at 100%, with effect sizes increasing from negligible to large and ICC remaining moderate (Table 4). While sex did not significantly moderate HR differences in tests across stages (Table 2), a significant sex difference in ICC was observed at baseline (ΔICC: 0.355, *P* = 0.002), but not during exercise (Table 4). Bland–Altman analysis for HR revealed that 95% LoA widened progressively with increasing test intensity in both sexes (Figure 2). Among adolescent males, 95% LoA were −16 to 27 bpm at 25%, −18 to 37 bpm at 50% and −8 to 36 bpm at 100% test completion. Similarly, among females, 95% LoA expanded from −24 to 24 bpm, −16 to 33 bpm and −7 to 32 bpm at 25%, 50% and 100% test completion, respectively.

**TABLE 4:** Cohen's *d* and ICC by sex, and analysis of sex moderation (difference in differences) for HR and MCAv for 30 adolescent participants (15F:15M, aged 14–17) who completed both the CARE test and CCCT

		Male				Female				Difference in differences		
Parameter	Comparison	Cohen's *d*	Cohen's d level	ICC	ICC level	Cohen's *d*	Cohen's *d* level	ICC	ICC level	Cohen's *d*	ICC	*P*‐value
**HR (bpm)**	**Baseline**	0.025	negligible	0.894	good	0.277	small	0.539	moderate	−0.235	0.355	0.002^*^
	**25%**	0.445	small	0.547	moderate	0.012	negligible	0.517	moderate	0.302	0.030	0.772
	**50%**	0.763	moderate	0.460	poor	0.724	moderate	0.518	moderate	0.058	−0.058	0.579
	**75%**	0.700	moderate	0.387	poor	1.159	large	0.507	moderate	−0.236	−0.120	0.248
	**100%**	1.029	large	0.382	poor	1.161	large	0.564	moderate	−0.057	−0.182	0.092
**MCAv (cm/s)**	**Baseline**	0.043	negligible	0.840	good	0.123	negligible	0.950	excellent	−0.053	−0.109	0.224
	**25%**	0.208	small	0.658	moderate	0.181	negligible	0.675	moderate	0.020	−0.017	0.851
	**50%**	0.325	small	0.607	moderate	0.492	small	0.661	moderate	−0.106	−0.054	0.570
	**75%**	0.695	moderate	0.517	moderate	0.577	moderate	0.584	moderate	0.111	−0.067	0.498
	**100%**	0.589	moderate	0.522	moderate	0.410	small	0.619	moderate	0.157	−0.097	0.325

Data were normalised to percentage of test completion to account for variations in test duration between the CARE test and CCCT. ^*^Significance (*P *< 0.05). Abbreviations: CARE test, Calgary Adapted aRm Ergometer Test; CCCT, Calgary Concussion Cycle Test; HR, heart rate; ICC, intraclass correlation; MCAv, middle cerebral artery blood velocity.

In males, mean MCAv differences between the CCCT and CARE increased from 2.6 cm/s (95% CI: –2.7, 7.9) at 25% to 9.9 cm/s (95% CI: 2.8, 16.9) at 75% and were 8.3 cm/s (95% CI: 1.3, 15.3) at 100% test completion (Table 2). ICC values remained moderate, while effect sizes progressed from small to moderate (Table 4). Among females, MCAv differences between protocols were 2.3 cm/s (95% CI: –3.0, 7.5) at 25%, 7.7 cm/s (95% CI: 1.3, 14.1) at 75% and 5.3 cm/s (95% CI: –0.6, 11.2) at 100% test completion. Cohen's *d* changed from negligible to moderate to small at 25%, 75% and 100% test completion, respectively, while ICCs remained moderate (Table 4). No significant sex moderation was observed for MCAv mean differences or ICC differences at any stage (Tables 2 and 4). Bland–Altman analysis showed relatively stable lower 95% LoA across test completion in males, with increasing upper LoA at increased intensities: −19.9 to 25.1 cm/s, −22.4 to 30.9 cm/s and −21.6 to 38.3 cm/s at 25%, 50% and 100% test completion, respectively (Figure 3). In females, both lower and upper 95% LoA remained relatively consistent across stages, ranging from −21.4 to 25.9 cm/s at 25%, −19.9 to 32.3 cm/s at 50% and −20.4 to 31.0 cm/s at 100% test completion (Figure 3).

### 3.2 Respiratory responses ($P_{\mathrm{ETC}O_{2}}$, relative ${\dot{V}}_{O_{2}}$ and ${\dot{V}}_{E}$)

For $P_{\mathrm{ETC}O_{2}}$, mean differences between CCCT and CARE increased progressively through 75% of test completion in both sexes before declining at 100% (Figure 4). For males, differences rose from 1.8 Torr (95% CI: 0.4, 3.2) at 25% to 3.1 Torr (95% CI: 1.5, 4.7) at 75%, then declined to −0.8 Torr (95% CI: −3.2, 1.5) at 100% test completion (Table 3). In females, differences ranged from 2.0 Torr (95% CI: 0.5, 3.5) at 25% to 4.3 Torr (95% CI: 2.8, 5.8) at 75%, then decreased to 3.0 Torr (95% CI: 0.9, 5.1) at 100% test completion. Effect sizes changed from moderate to large in females and moderate to small in males as test completion progressed, while ICC decreased from moderate to poor in both sexes (Table 5). Sex moderated $P_{\mathrm{ETC}O_{2}}$ differences only at 100% completion (difference in differences [DiD]: −3.8 Torr, 95% CI: −6.9, −0.6), while a significant ICC difference was observed at baseline (ΔICC: –0.184, *P* = 0.042) but not during exercise. (Tables 3 and 5). Among males, Bland–Altman 95% LoA ranged from −4.5 to 8.1 Torr at 25%, ‐3.1 to 9.2 Torr at 75% and −7.7 to 6.0 Torr at 100% test completion (Figure 5). In females, a similar range compared to males was observed at 25% and 50% test completion: −4.3 to 8.3 Torr and −2.2 to 10.8 Torr, respectively, while at 100% test completion the 95% LoA was −2.8 to 8.7 Torr (Figure 5).

**TABLE 5:** Cohen's *d* and ICC by sex, and analysis of sex moderation (difference in differences for Cohen's *d* and ICC) for $P_{\mathrm{ETC}O_{2}}$, ${\dot{V}}_{E}$, and ${\dot{V}}_{O_{2}}$ for 30 adolescent participants (15F:15M, aged 14–17) who completed both the CARE test and CCCT

		Male				Female				Difference in differences		
Parameter	Comparison	Cohen's *d*	Cohen's *d* level	ICC	ICC level	Cohen's *d*	Cohen's *d* level	ICC	ICC level	Cohen's *d*	ICC	*P*‐value
$P_{\mathrm{ETC}O_{2}}$ **(Torr)**	**Baseline**	0.050	negligible	0.231	poor	0.341	small	0.415	poor	−0.244	−0.184	0.042^*^
	**25%**	0.592	moderate	0.565	moderate	0.654	moderate	0.537	moderate	−0.055	0.027	0.788
	**50%**	0.965	large	0.676	moderate	1.234	large	0.665	moderate	−0.197	0.012	0.903
	**75%**	0.977	large	0.515	moderate	1.399	large	0.540	moderate	−0.279	−0.025	0.811
	**100%**	0.206	small	0.323	poor	0.801	large	0.372	poor	−0.697	−0.049	0.595
${\dot{V}}_{E}$ **(L/min)**	**Baseline**	0.034	negligible	0.508	moderate	0.146	negligible	0.738	moderate	−0.120	−0.230	0.053
	**25%**	0.657	moderate	0.627	moderate	0.043	negligible	0.662	moderate	0.441	−0.035	0.713
	**50%**	1.024	large	0.491	poor	0.499	small	0.637	moderate	0.440	−0.146	0.157
	**75%**	1.471	large	0.264	poor	0.520	moderate	0.411	poor	0.823	−0.147	0.113
	**100%**	1.837	large	0.059	poor	1.026	large	0.113	poor	0.890	−0.054	0.116
**Relative** ${\dot{V}}_{O_{2}}$ **(ml/kg/min)**	**Baseline**	0.148	negligible	0.913	excellent	0.023	negligible	0.887	good	0.088	0.026	0.633
	**25%**	1.801	large	0.199	poor	1.253	large	0.256	poor	0.524	−0.057	0.407
	**50%**	2.021	large	0.116	poor	1.667	large	0.154	poor	0.431	−0.038	0.416
	**75%**	1.862	large	0.048	poor	1.551	large	0.083	poor	0.543	−0.035	0.171
	**100%**	2.067	large	0.027	poor	1.579	large	0.050	poor	0.738	−0.023	0.140

Data were normalised to percent of test completion to account for variations in test duration between the CARE test and CCCT. ^*^Significance (*P *< 0.05). Abbreviations: CARE test, Calgary Adapted aRm Ergometer Test; CCCT, Calgary Concussion Cycle Test; ICC, intraclass correlation; $P_{\mathrm{ETC}O_{2}}$, partial pressure of end‐tidal carbon dioxide; ${\dot{V}}_{E}$, minute ventilation; ${\dot{V}}_{O_{2}}$, relative volume of oxygen consumption.

For ${\dot{V}}_{E}$, values on the CCCT were consistently higher than the CARE test across test completion in males, with increasing differences from 3.5 L/min (95% CI: 1.2, 5.8) at 25% to 31.9 L/min (95% CI: 19.9, 44.0) at 100% test completion (Table 3). Cohen's *d* ranged from moderate to large and ICC dropped to poor at 50% test completion (Table 5). Among females, ${\dot{V}}_{E}$ differences were not significant at 25% or 75% but were significance at 50% (2.6 L/min, 95% CI: 0.4, 4.9) and 100% (12.9 L/min, 95% CI: 4.4, 21.3). Cohen's *d* increased from negligible to large with poor ICC as test completion progressed. Sex significantly moderated ${\dot{V}}_{E}$ mean differences at 25% (DiD: 3.3 L/min, 95% CI: 0.1, 6.4, *P* = 0.043) and 100% (DiD: 19.1 L/min, 95% CI: 4.3, 33.8, *P* = 0.011), though ICC differences were not significant different between sexes (Tables 3 and 5). Bland–Altman analysis demonstrated progressively widening 95% LoA for ${\dot{V}}_{E}$ with increasing test completion in both sexes (Figure 6). Among males, 95% LoA ranged from −6.5 to 13.5 L/min at 25%, −6.9 to 19.1 L/min at 50% and 3.1 to 60.8 L/min at 100% test completion. In females, lower 95% LoA were relatively comparable to males while upper 95% LoA were generally smaller: −9.5 to 9.9 L/min, −6.6 to 11.9 L/min and −5.7 to 31.4 L/min at 25%, 50% and 100% test completion, respectively.

Lastly, ${\dot{V}}_{O_{2}}$ differences in males were consistently greater during CCCT compared to CARE, increasing from 5.4 mL/kg/min (95% CI: 3.4, 7.3) at 25% to 16.8 mL/kg/min (95% CI: 11.1, 22.6) at 100% (Table 3). Large effect sizes and poor ICC were observed across all exercise comparisons (Table 5). Similarly, females had significant ${\dot{V}}_{O_{2}}$ differences, ranging from 3.3 mL/kg/min (95% CI: 1.7, 4.9) at 25% to 9.4 mL/kg/min (95% CI: 5.2, 13.5) at 100%, with large effect sizes and poor ICC throughout (Tables 3 and 5). Sex significantly moderated ${\dot{V}}_{O_{2}}$ mean differences between tests, but only at 100% test completion (DiD: 7.4 mL/kg/min, 95% CI: 0.4, 14.5, *P* = 0.040), while no significant ICC differences were found (Tables 3 and 5). Similar to the other physiological variables, Bland–Altman analysis for ${\dot{V}}_{O_{2}}$ showed widening 95% LoA for both males and females (Figure 7). Within males, 95% LoA expanded from −1.1 to 11.2 mL/kg/min at 25%, 1.6 to 21.0 mL/kg/min at 75% and 1.1 to 32.6 mL/kg/min at 100% test completion. In contrast, it appeared females displayed narrower ranges at higher intensities, with 95% LoA of −2.7 to 9.2 mL/kg/min, −3.2 to 17.5 mL/kg/min and −0.1 to 18.9 mL/kg/min at 25%, 50% and 100%, respectively.

## 4 DISCUSSION

The present investigation compared the physiological responses observed during an upper body specific graded exercise test (CARE) test to be used for the management of concussion with a previously validated, lower body specific protocol (CCCT) in a sample of healthy, athletic, non‐disabled male and female adolescents. This study builds off previous work which compared the CARE test to the CCCT in healthy, non‐disabled adults, highlighting the utility of ACE for measuring symptom response profiles post‐concussion to further enhance the accessibility and inclusivity of concussion care (Smirl et al., 2026). The key findings from this investigation revealed physiological responses were most comparable at low‐to‐moderate intensity levels (25% and 50% test completion), a similar finding compared to the study within adult participants (Figures 1 and 4) (Smirl et al., 2026). At higher intensities (75% and 100%), physiological differences between protocols increased for both adolescent males and females, and sex was found to moderate the relationship between tests for ventilatory parameters ($P_{\mathrm{ETC}O_{2}}$, ${\dot{V}}_{E}$ and ${\dot{V}}_{O_{2}}$) (Table 3). Specifically, adolescent males demonstrated greater peak ${\dot{V}}_{E}$ and ${\dot{V}}_{O_{2}}$ during the CCCT relative to the CARE test, while females displayed smaller mean differences between tests (Figure 4 and Table 3). While the CARE test elicited lower absolute values compared to the CCCT at increased intensity levels for both males and females, robust physiological response profiles were observed for each variable, typical for what is expected from the larger exercise physiology literature (Larsen et al., 2016; McArdle et al., 2010; Smith & Ainslie, 2017). Notably, the similarity of responses at low‐to‐moderate intensities offers further support to the use of ACE following concussion. At these intensity levels, which are most commonly used in clinical post‐concussion exertion testing (Leddy et al., 2018), male and female adolescents showed comparable physiological profiles, with no sex‐based moderation of test responses (Tables 2 and 3). These trends were further supported by Bland–Altman analysis, which generally demonstrated narrower 95% limits of agreement at lower intensities, suggesting improved individual‐level agreement between tests in the clinically relevant low‐to‐moderate exertion range. These findings reinforce CARE as a potential upper‐body alternative for assessing exercise capacity and symptom provocation in adolescents post‐concussion who cannot perform lower‐body exercise (i.e., Para athletes, concurrent injury) or who may prefer or perform upper‐body dominant forms of exercise (i.e., swimmers, rowers).

### 4.1 Systemic physiological responses of ACE and leg cycling

ACE has been evaluated for its ability to offer numerous health and cardiovascular benefits for individuals not able to perform lower body exercise or for those who participate in predominately upper body activities (Chiou et al., 2022). While previous studies have highlighted differences in physiological responses between ACE and leg cycling in adults (Larsen et al., 2016), to the authors knowledge, this study is the first to compare these responses to volitional fatigue in adolescents. Previous differences in peak ${\dot{V}}_{O_{2}}$, HR and ${\dot{V}}_{E}$ observed in adults are echoed in the current investigation (Smirl et al., 2026). Namely, differences were augmented at increased exercise intensity, with the largest differences in systemic physiological responses observed at 100% of test completion (Hill et al., 2019; Larsen et al., 2016; Mitropoulos et al., 2017; Muraki et al., 2004; Smirl et al., 2026) (Tables 2 and 3). These differences likely reflect the smaller active muscle mass and lower sympathetic stimulation associated with ACE (Schneider et al., 2002), and the higher proportion of type II muscle fibres in the arms, which contribute to earlier anaerobic recruitment and reduced metabolic efficiency (Muraki et al., 2004; Orr et al., 2013; Schneider et al., 2002). Interestingly, peak ${\dot{V}}_{E}$ and ${\dot{V}}_{O_{2}}$ differences between tests were greater in males than females, likely driven by similar peak responses on the CARE test and higher peak values in males during the CCCT (Figure 4). This may reflect the lower aerobic demands of ACE, which recruits smaller muscle groups and relies more heavily on anaerobic metabolism potentially minimising the sex‐based differences in aerobic capacity typically observed during leg cycling (Schneider et al., 2002; Welde et al., 2020). Furthermore, peak ${\dot{V}}_{O_{2}}$ differences between leg cycling and ACE in the present adolescent sample (males: 16.8 mL/kg/min; females: 9.4 mL/kg/min) were comparable to those reported in the adult study (11.8 mL/kg/min) (Smirl et al., 2026) and a 2016 meta‐analysis in adults (mean difference: 12.5 mL/kg/min) (Larsen et al., 2016), suggesting that systemic physiological differences between modalities are already evident in adolescence and may be more pronounced in males. These findings reinforce the need to interpret exertional responses in the context of both sex and exercise modality when evaluating post‐concussion exercise tolerance in adolescents.

Adolescent male and female participants displayed similar HR response profiles, and mean difference comparisons between CARE and CCCT were not moderated by sex (Figures 1 and 2 and Table 2). HR mean differences were most pronounced at 100% test completion: 14 and 13 bpm lower during CARE in males and females respectively (Table 2). These mean differences were greater than those observed in adults who performed both CARE and CCCT (mean difference: 8 bpm lower during CARE) (Smirl et al., 2026). Greater HR differences observed in adolescents compared to adults may be attributable to adolescents’ ongoing development in neuromuscular coordination and motor control (Casamento‐Moran et al., 2018; Falk et al., 2009). Related to SRC, the similarity of HR responses between tests, especially at low‐to‐moderate intensities (25% and 50%) is relevant, as the HR response post‐concussion exertion testing is important for prescribing post‐injury aerobic exercise intensity (Leddy et al., 2023). Therefore, HR response similarities for both male and female adolescent athletes performing the CARE test and CCCT offer further support towards the utility of ACE for prescribing sub‐symptom threshold aerobic exercise in populations who cannot complete the BCTT or CCCT (i.e., Para athletes, lower body injury). Additionally, the widening 95% LoA for HR for males and females with increasing test completion suggests that considerable individual‐level variability exists between tests. This variability likely reflects individual differences in muscle mass distribution, training history and fitness level which may have differentially altered the response to upper body vs. lower body resistance exercise. Future research is necessary to explore HR responses in Para athletes and non‐disabled individuals with greater upper‐body training experience. Furthermore, due to the mean difference in maximum HR achieved, it is possible the CARE test may have to adopt a lower HR cutoff in a clinical setting compared to the typical 180 bpm cutoff recommended in the BCTT protocol (Leddy & Willer, 2013). However, research comparing the HR response in athletes more familiar with upper body exercise, especially Para athletes, is warranted before further recommendation to update concussion treatment guidelines can be made (Patricios et al., 2023).

### 4.2 Autonomic dysfunction, exercise intolerance and cerebrovascular considerations

Autonomic dysfunction, a common post‐concussion sequela, is related to alterations in cardiovascular, respiratory and cerebrovascular control (Esterov & Greenwald, 2017). These impairments are believed to underlie exercise intolerance in affected individuals, which in turn has been associated with delayed recovery (Haider et al., 2022; Leddy et al., 2018; Neill et al., 2025; Pelo et al., 2023). In the present study, both adolescent males and females exhibited robust MCAv responses during the CARE test, with comparable trajectories to the CCCT at low‐to‐moderate intensities that were not moderated by sex (Figure 1 and Table 2). Impairments in the ability of the cerebrovasculature to modulate exercise‐induced changes in blood pressure may exacerbate symptoms (Worley et al., 2021). While MAP was not able to be recorded in the present study due to inadequate signal quality, previous reports have demonstrated robust blood pressure responses during ACE and leg cycling (Dias et al., 2022), with ACE often eliciting higher blood pressure at equivalent absolute workloads (Calbet et al., 2015). Accordingly, the smaller incremental wattage increases in the CARE test compared to the CCCT and the comparable test duration help promote a more gradual and physiologically appropriate rise in MAP, ensuring that blood pressure increases in proportion to overall cardiovascular demand.

Furthermore, concussion has been associated with increased cerebrovascular reactivity, associated with increased CBF relative to exercise intensity (Albalawi et al., 2017; Howell et al., 2021; Neill et al., 2024). This dysregulation may contribute to the onset or worsening of symptoms such as headache, dizziness and pressure in the head during exertion (Albalawi et al., 2017; Howell et al., 2021). In addition, impairments in ventilatory control post‐concussion have been reported, characterised by relative hypoventilation and elevated arterial carbon dioxide levels (Leddy et al., 2018). These ventilatory and associated cerebrovascular consequences may further amplify cerebral hyperaemia during exercise and exacerbate symptom burden, potentially contributing to exercise intolerance (Leddy et al., 2018). In the present study, the CARE test evoked the expected bell‐shaped MCAv response to increasing exercise intensity in both male and female adolescents (Smith & Ainslie, 2017) (Figure 1). Although absolute MCAv values were lower during CARE compared to the CCCT, this pattern mirrored the attenuated $P_{\mathrm{ETC}O_{2}}$ response also observed, aligning with results previously reported in adults (Figures 1 and 4) (Smirl et al., 2026). This is notable due to the paucity of research exploring the cerebrovascular response to varying exercise modalities in adolescents. Relevant to adolescent SRC, headaches and dizziness are two of the most common symptoms reported by adolescents (Ingram et al., 2025) and commonly exacerbated during exertion testing (Howell et al., 2021; Leddy et al., 2018). ACE may enable exercise at intensity levels limited by larger increases in MCAv and $P_{\mathrm{ETC}O_{2}}$ and subsequent symptoms from lower body exercise while still providing an appropriate cerebrovascular challenge to assess exercise (in)tolerance for those not capable of performing lower body specific protocols. Moreover, while HR responses were similar between tests at 25% and 50% test completion, HR at symptom exacerbation for exercise intolerant individuals may potentially be lower during ACE due to reduced systemic strain from smaller active muscle mass (Larsen et al., 2016). Although speculative, this highlights the need to establish sub‐symptom thresholds and physiological markers of exercise intolerance in concussed adolescents using the CARE test. Altogether, the present study offers further support of the CARE protocol's potential to expand access to safe and inclusive exertion testing; however, evaluation in Para athletes and those more accustomed to upper‐body exercise is necessary before clinical recommendations can be made.

### 4.3 Clinical implications

Adolescents represent a substantial proportion of individuals affected by, and at risk for, SRC (Black et al., 2021; Zhang et al., 2016). Despite increasing support for the use of exertion testing and sub‐symptom threshold aerobic exercise in adolescent concussion management (Patricios et al., 2023), limited research exists characterising the full physiological responses to exertion protocols in this population. Furthermore, current protocols such as the BCTT and CCCT require lower‐body function and may be impractical for adolescents with limited functional capacity, concurrent musculoskeletal injury and/or excessive concussion‐related symptoms (Ryan et al., 2024). This gap is particularly evident in adolescent Para sport, where athletes may face a greater risk of concussion due to the nature of adapted sports but remain severely under‐represented in SRC research (Sobry et al., 2022). As such, a call‐to‐action was made regarding greater representation of research directed towards SRC in Para athletes during the latest concussion consensus statement (Patricios et al., 2023) and reiterated in the first position statement on concussion in Para sport (Weiler et al., 2021). Even with these *calls for action*, there has unfortunately been minimal research into the challenges faced by Para athletes when they are navigating concussion recovery and specifically the need for an exertion test for people with lower limb impairments (Ryan et al., 2024). Furthermore, it has been shown Para athletes report having both a higher number of symptoms endorsed and a greater total symptom severity burden, further highlighting the need for improved concussion management strategies within this population (Lexell et al., 2021). The CARE test helps address this critical clinical gap by offering an upper‐body‐specific physiological alternative exertion protocol that can be safely completed by adolescents and tailored towards concussion management in adapted populations (Smirl et al., 2026). The present findings suggest the CARE test elicits appropriate physiological responses for assessing exercise tolerance post‐concussion in adolescents and may assist in identifying sub‐symptom threshold HR targets for clinically guiding post‐concussion management via exercise prescription, particularly in those unable to perform lower‐body protocols. Notably, Bland–Altman analyses revealed relatively broad 95% LoA for each outcome which generally widened with increased test completion (Figures 2, 3 and 5, 6, 7). In a clinical context, where exertion testing and exercise prescriptions are administered in a highly individualised manner, such variability reinforces the need for tailored interpretations of exertion test data instead of a one‐size‐fits‐all approach. Importantly, despite variability in physiological agreement, maximal RPE was comparable at test termination across protocols, suggesting similar attainment of participants’ subjective maximal effort. While individual variability in the present study may partially be attributed to limited participant familiarity with upper‐body exercise, such variability may influence exercise tolerance and symptom exacerbation thresholds when applied in concussed populations. This underscores the importance of future research addressing the response similarities within populations more familiar with upper body exercise and Para athletes. Finally, this work builds on research comparing CARE to CCCT in non‐disabled adults, representing necessary preliminary steps in the validation process of an upper‐body specific, post‐concussion exertion test to ensure accessible and inclusive protocols exist for athletes of all abilities (Smirl et al., 2026).

### 4.4 Sex‐specific considerations

Females may be at increased risk of concussion (Bretzin et al., 2018), delayed recovery following concussion (Putukian et al., 2023), developing PSaC (Zemek et al., 2016) and being intolerant to exercise (Neill et al., 2025). Sex differences in exercise intolerance may explain recovery differences (Neill et al., 2025) and may relate to differences in CBF regulation. Sex‐based differences in CBF and cerebrovascular regulation have been documented in adolescents, with males typically showing greater reductions in CBF during puberty and females demonstrating a rebound increase during mid‐to‐late puberty (Satterthwaite et al., 2014). The divergent cerebrovascular developmental trajectories support the importance of assessing the influence of sex to enhance the interpretability and generalisability of findings. Moreover, females exhibit higher CBF during rest and exercise compared to males (Douglas et al., 2024), have higher absolute cerebrovascular reactivity than males (Burma et al., 2025), and display a lower buffering to systolic blood pressure than males (Johnson et al., 2024). Thus, while females have improved cerebrovascular regulation when healthy, they may be more vulnerable to increased CBF during exercise following concussion, given higher resting CBF levels and regulation which tends to elicit larger increases in CBF during exercise. Furthermore, evidence where blood samples were used to confirm self‐report menstrual cycle phase report evidence that CBF levels might undergo transient changes across the menstrual cycle, due to fluctuations in oestrogen and progesterone (Peltonen et al., 2016). Conversely, menstrual cycle phase did not alter the cerebrovascular response to exercise in a study using urinary ovulation measures to estimate menstrual phase (Persaud et al., 2024). A recent study did, however, report that roughly 25% of adolescents’ following concussion self‐report two or more abnormal menstrual patterns, defined by intermenstrual interval of <21 days or >35 days and/or bleeding duration <3 days or >7 days whereas only 5% of those with orthopaedic injury reported the same (Snook et al., 2017). Thus, while this study did not collect details on menstrual cycle phase, further investigation on the influence of menstrual (dys)function following concussion on cerebrovascular function and exercise tolerance is needed, and ideally this research should employ objective measures of menstrual function and cycle phase to reduce bias associated with self‐report techniques or estimations (Elliott‐Sale et al., 2025). In the present study, male and female adolescents exhibited largely comparable response profiles across both exertion protocols. HR and MCAv responses between tests were not moderated by sex at any comparison time point while $P_{\mathrm{ETC}O_{2}}$ differences were only moderated by sex at 100% test completion. At higher intensities (75% and 100% test completion), sex significantly moderated the ventilatory response relationship between tests, with males displaying greater ${\dot{V}}_{E}$ and ${\dot{V}}_{O_{2}}$ differences compared to females. This aligns with existing literature highlighting that sex‐based differences in musculoskeletal development become more pronounced post‐puberty (Nuzzo & Pinto, 2025), supported by the present investigation in which the majority of participants were estimated to be post‐pubertal. Of particular relevance to SRC, the expected bell‐shaped MCAv response was elicited in both sexes during the CARE and CCCT, with comparable MCAv elevations observed during mild‐to‐moderate exercise intensities. Bland–Altman plots for each variable revealed individual variability in agreement between modalities, with 95% LoA typically widening with test completion in both sexes. At low‐to‐moderate intensities, 95% LoA were similar between sexes suggesting that while sex did not significantly influence overall agreement, individual variability between upper‐ and lower‐body exertion should be acknowledged and assessed in future research and clinical implementation. Collectively, these findings provide additional support for the clinical utility of both the CARE and CCCT protocols in assessing exercise tolerance and concussion symptomatology in male and female adolescent athletes. Moreover, while not the primary objective, this study also represents the first evaluation of the CCCT in an adolescent population, offering physiologically grounded support for the use of cycling‐based exertion testing in both male and female adolescents post‐concussion.

### 4.5 Limitations and future directions

This investigation is limited by the characteristics of the study sample. Namely, participants were non‐disabled, largely classified as post‐pubertal, and generally physically fit. While all female participants were estimated to be post‐pubertal using Khamis–Roche and Moore maturity offset methods, age of menarche onset was not captured. As maturation status and pubertal development influence cerebrovascular, cardiovascular, ventilatory and metabolic responses to exercise during adolescence, the absence of specific menarche data and the small number of pubertal/circa‐PHV participants limits the ability to fully account for biological maturation as a contributor to the observed physiological responses (Armstrong & Welsman, 2020; Armstrong et al., 2015; Satterthwaite et al., 2014). Future work comparing exertion tests in younger participants, less fit participants and Para athletes (adolescents and adults) is necessary to enhance clinical generalisability. Future research should also include concussed and non‐concussed participants (non‐disabled and Para athletes) to better understand how the intersection of injury and impairment status may influence test responses. In addition, the study is limited by the use of TCD, which assesses MCAv as an index of CBF under the assumption vessel diameter remains constant (Ainslie & Hoiland, 2014). While minimal changes in vessel diameter are observed around eucapnia (Ainslie & Hoiland, 2014), during the later stages of the exertion tests, large decreases in $P_{\mathrm{ETC}O_{2}}$ may have caused vessel constriction potentially underestimating CBF near maximum exertion. Despite this, any underestimation of global CBF would be non‐differential, and is unlikely to influence agreement between exertion tests. TCD remains a common method to assess the cerebrovasculature during exercise (Neill et al., 2024; Willie et al., 2011). Lastly, participants’ limited familiarity with ACE may have contributed to increased individual response variability and a potential overestimation of physiological differences between the CARE test and the CCCT at higher intensities near volitional fatigue. The novelty of ACE may have reduced neuromuscular efficiency, causing participants to terminate the test prior to reaching true physiological exhaustion (Waldron et al., 2016). However, both tests elicited robust response profiles and RPE at test termination was 20 for CARE and CCCT, suggesting participants neared maximal effort.

### 4.6 Conclusion

This study demonstrated physiological comparability between the CARE test CCCT in an adolescent population. The CARE test produced lower HR, MCAv, $P_{\mathrm{ETC}O_{2}}$, ${\dot{V}}_{O_{2}}$ and ${\dot{V}}_{E}$ compared to the CCCT with differences more pronounced near volitional fatigue. Similar differences were observed in male and female adolescent participants at low‐to‐moderate intensity. Thus, the CARE test represents an appropriate, upper body specific, physiological alternative protocol for assessing symptomology and exercise capacity following concussion in adolescents. Future research should evaluate CARE test responses in Para athletes and individuals unable to perform lower‐body protocols, compared to those who can complete both modalities before clinical implementation of the CARE protocol Supporting Information.

## AUTHOR CONTRIBUTIONS

Joshua J. Burkart and Jonathan D. Smirl conceptualised and designed the study. Jonathan D. Smirl, Joshua J. Burkart, Matthew G. Neill, Tegan Wilder, Sarah Johns, Joseph Carere, performed the experiments. Joshua J. Burkart and Jean‐Michel Galarneau conducted the data analysis. Joshua J. Burkart and Jonathan D. Smirl wrote the manuscript. Matthew G. Neill, Jean‐Michel Galarneau, Tegan Wilder, Sarah Johns, Joseph Carere, John J. Leddy, Mohammad N. Haider, William M. Adams, Cheri Blauwet, Chantel T. Debert and Carolyn A. Emery contributed to editing and revising the manuscript. All authors have approved the final version of the manuscript and agree to be accountable for all aspects of the work, ensuring that any questions related to the accuracy or integrity of any part of the work are appropriately investigated and resolved. All contributing and corresponding authors qualify for authorship, and all those who qualify for authorship are listed.

## CONFLICT OF INTEREST

None declared.

## GENERATIVE AI STATEMENT

The authors confirm that no artificial intelligence tools, including large language models (LLMs), were used in the drafting or revision of this manuscript. All content was conceived, written, and approved solely by the authors.

## Supporting information



Supporting information

## Data Availability

Data from the article are available upon reasonable request to the corresponding author (J.J.B).
